# Perceptions of Altered Smile Esthetics: A Comparative Evaluation in Orthodontists, Dentists, and Laypersons

**DOI:** 10.1155/2016/7815274

**Published:** 2016-09-28

**Authors:** Amjad Al Taki, Mohammed Khalesi, Muftah Shagmani, Islam Yahia, Fatma Al Kaddah

**Affiliations:** Private Practice, Dubai, UAE

## Abstract

*Objective*. The current investigation was proposed to determine the impression of trained dental professionals and laypeople towards the modified smile esthetics.* Materials and Methods*. Twenty-six images were randomized in a survey and graded according to attractiveness by the orthodontists, general dentists, and laypeople. Photographs of gingival display, midline diastema, central incisor crown length, and lateral incisor crown width were manipulated with five minor changes in each. For smile arc and buccal corridor, two major changes were incorporated besides the ideal photograph. One-way ANOVA and Post Hoc analysis of the responses were measured for each group.* Results*. Most evaluators opined that the ideal smile in each category was the most acceptable. Orthodontists were more perceptive and exacting in accepting variations in the smile arc and buccal corridors. Dental professionals and laypeople indicated that either complete absence or a 0.5 mm of alterations in a gingival display, midline diastema, and crown length makes a smile beautiful and pleasant. Changes in crown width were not perceivable by all the three groups.* Conclusion*. Eastern Arabic laymen are more conscious about alterations in gingival display, midline diastema, and crown length in their smile. Hence, the orthodontist should pay attention to these factors during any orthodontic treatment.

## 1. Introduction

All humans desire esthetically pleasing features and the smile is one of the most sought features. Smile analysis is an integral part of the overall facial analysis carried out by dental specialties. Assessing patient's smile allows the clinician to see what needs to be done, what can be done, and what should be accepted. A smile analysis includes assessing variables such as the amount of the incisors and gingiva show upon smiling, the smile arc (parallelism between the maxillary incisal edges and the lower lip), tooth proportions, gingival height and contours, relationship between the dental midline and facial midline, and tooth shade and color [1]. An esthetically pleasing smile is dependent on the harmony and symmetry between these variables. Currently, the demand for smile esthetics is growing; being thus, various smile variables need to be taken into consideration [2].

The perception of smile esthetics is subjective and is influenced by personal experiences and social environment [3]. Further, numerous studies have concluded that dental professional and general population differ considerably in their preferences for smile esthetics [4, 5]. Moreover, among the dental professionals, the orthodontists are more analytical than the general dentist. This is due to the special training of orthodontist to observe and evaluate features that do not seem to influence the general dentist and the public.

Different ethnic populations have their preferences for smile esthetics. Comparison between US Caucasians, US American Asian Indians, and Indians residing in India revealed that considerable differences exist between the three groups for certain smile variables [6]. Buccal corridor space was preferred by US Indians and Indians compared to US Caucasians. Similarly, ideal and minimum smile arc were chosen by the US Indians and Indians compared to the US Caucasians. Even though maximum and ideal gingival display were similar between all the three groups, a minimal gingival display was preferred by the US Indians and Indians. Nevertheless, minor discrepancies existed between the US Indians and Indians residing in India. In another similar study, McLeod et al. analyzed the smile variables (such as buccal corridor, gingival display, occlusal cant, maxillary midline to face discrepancy, and lateral central gingival discrepancy) between the US and Canadian populations [7]. They observed clinically remarkable differences in all variables, except buccal corridor. Moreover, the Canadians were more discerning than the US population. Notable variation also existed between German, Russian, and Turkish population towards a perception of smile variables [8]. Therefore, regional studies on evaluation of smiles esthetics are necessary. Few studies are reported on preferences of various smile variables in populations of northern and central regions of Arabian Peninsula [9, 10]. However, till date no study has been published which assessed the smile esthetics in the eastern region of Arabian Peninsula.

A smile which appears beautiful in the first instance might not be in the second instance. This plays a significant role in determining the threshold level of acceptable deviations in different variables responsible for making a smile pleasing and attractive. Most of the studies assessing the smile esthetics have assessed the variables only once and have determined the threshold levels based on them [6–10]. This might not be the true representation of the threshold. Hence, scoring the same smile variable more than once might show the actual threshold level.

The present study aimed at evaluating the differences in perceiving factors that affect the smile esthetics, among orthodontists, general dentists, and laypersons of Dubai (UAE), and testing the hypotheses that (1) orthodontists are more perceptive than general dentists and laypeople in detecting esthetic discrepancies; (2) laypeople are less perceptive than general dentists and orthodontists in detecting esthetic discrepancies; and (3) orthodontists are more critical than dentists and so are dentists compared to laypersons in detecting esthetic discrepancies.

## 2. Materials and Methods

### 2.1. Samples

In a cross-sectional study, a total of 110 evaluators participated, including 28 orthodontists, 35 general dentists, and 47 laypersons. The mean age of laypersons group was 32 ± 9.53 years. The dentist group included general practitioners, with a mean age of 34 ± 7.81 years, while the orthodontist group had a mean age of 36 ± 8.62 years. The majority of orthodontists and general dentists were males, with an average professional experience of more than 8 years. Available laypersons were contacted, and those with dental affiliations were excluded. The majority of laypersons were males and college educated.

### 2.2. Variables and Measurements

Orthodontist, dentist, and laypersons examined and valued six different esthetic variables to test our three hypotheses. The photographs showed the smile alone, excluding other facial structures, to minimize any confounding factors. Moreover, only female smiles were used, and similar skin tones were chosen. The smile features in the photographs were digitally modified by Adobe Photoshop software (Adobe Systems Inc., San Jose, CA). The modifications were purposely created to resemble a smile esthetic variation. After alteration, the images were condensed or enlarged to achieve an image size that represented the actual tooth size. A total of twenty-six digital photographs were used in this study.

The photographs were grouped into six sets, each representing an altered smile feature. The altered features were as follows: (1) smile arc, (2) buccal corridor, (3) gingival display (gummy smile), (4) midline diastema, (5) central incisor crown length, and (6) lateral incisor crown width. The changes were made incrementally. Photographs of gingival display, midline diastema, central incisor crown length, and lateral incisor crown width were manipulated with five minor changes in each and were evaluated twice. For smile arc and buccal corridor, two major changes were incorporated besides the ideal photograph and were measured only once. Two of the six (central incisor crown length, lateral incisor crown width without altering the crown length) were modified asymmetrically (unilaterally). All six alterations were selected after consultation with a clinically experienced orthodontist. These modifications were chosen based on their relatively high frequency in the population and their clinical significance to the smile.

#### 2.2.1. Smile Arc

**Figure 1:**
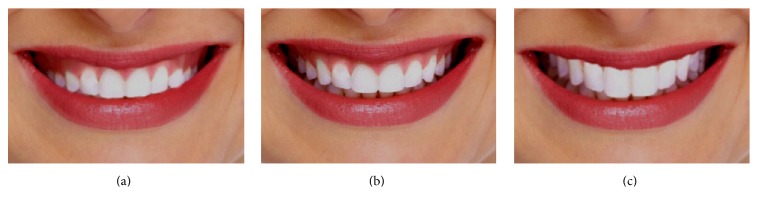
Illustration of alterations in the smile arc. (a) The smile arc is flat with a large gingival display in the posterior region compared to that in the anterior region, where the teeth arrangement aligns with the curvature of the lower lip. (b) An ideal smile arc that is parallel to the curvature of the lower lip. (c) Excessive smile arc causing the lower teeth to be displayed.

The photograph was modified by reversing and accentuating the curvature of the anterior teeth in relation to the curvature of the lower lip (see Figure 1).

#### 2.2.2. Buccal Corridor

**Figure 2:**
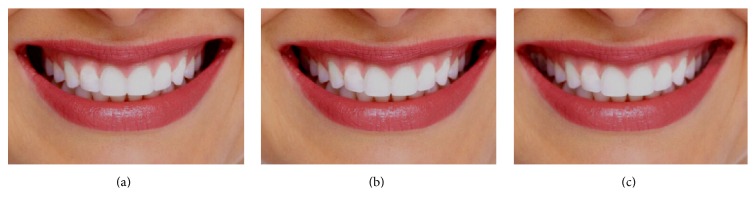
Representative photographs illustrating changes in buccal corridor. (a) Presence of excessive buccal corridors (dark corners) are increased in this image. (b) Control smile with ideal buccal corridors. (c) Broad smile with no buccal corridors (Hollywood smile).

The photograph was modified between the buccal surfaces of the maxillary teeth and the corners of the mouth (see Figure 2).

#### 2.2.3. Gingiva-to-Lip Distance

**Figure 3:**
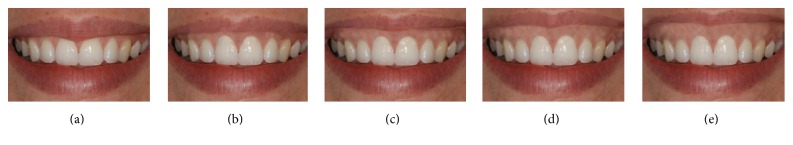
Photographs elucidating gingival display on the smile. Gummy smile images were obtained by an incremental raise in the gingiva-lip relationship. (a) Control, (b) 1 mm, (c) 2 mm, (d) 3 mm, and (e) 4 mm.

The gingiva-to-lip margin level (gingival show) was increased by 1 mm, to create a “gummy” smile. Modifications were based on the relationship of the upper lip with the gingival margin of the maxillary incisors (Figure 3).

#### 2.2.4. Midline Diastema

**Figure 4:**
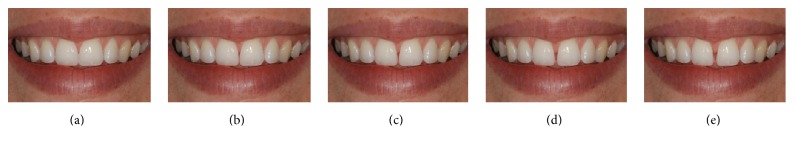
Photographs demonstrating modifications of a midline diastema. The alterations were done by an increment of 0.5 mm. (a) No alteration (control), (b) 0.5 mm midline diastema, (c) 1 mm diastema, (d) 1.5 mm diastema, and (e) 2 mm diastema.

A midline diastema was introduced between the maxillary central incisors by a 0.5 mm increment measured from interproximal contact point of the central incisors (Figure 4).

#### 2.2.5. Crown Length

**Figure 5:**
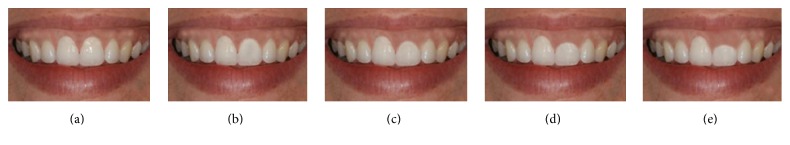
Photographs showing changes to the crown length of the maxillary left central incisors. Shortening of crown length was achieved by reducing the gingival margin height by 0.5 mm increments. (a) Control, (b) 0.5 mm, (c) 1.0 mm, (d) 1.5 mm, and (e) 2.0 mm.

The crown length of the maxillary left central incisor was altered by adjusting the level of the gingival margin, thereby shortening the length of the crown, in 0.5 mm increments. The reference point used for these measurements was the most superior point on the labial gingival margin of the patient's adjacent central incisor. The most common variation in incisor crown width is usually associated with the size of the maxillary lateral incisors; hence, the alterations of crown width were made to the maxillary lateral incisor (Figure 5).

#### 2.2.6. Crown Width

**Figure 6:**
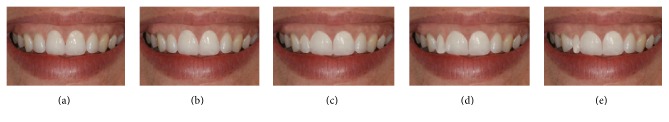
Photographs displaying alterations to maxillary lateral incisors crown width. Gingival margin maintained the same level, but the width of the maxillary right lateral incisors crown was decreased by an increment of 1 mm. (a) Control, (b) 1 mm, (c) 2 mm, (d) 3 mm, and (e) 4 mm, decrease in the width of the maxillary lateral incisors.

Symmetrical crown width alterations were made to the maxillary lateral incisors. The incisal edge was kept at the same level. The alteration was limited to the mesiodistal width of the lateral incisors, which was decreased by 1 mm (Figure 6).

Questionnaires were provided to the evaluators. The age, gender, and occupation were mentioned on the front page of the questionnaires. The photographs for each smile variable were grouped together in one page of the questionnaire; however, the sequence of the images was randomized. The attractiveness of the smile in the original image and in each of the modified images was assessed by the three groups and scored using a 5-point visual analog scale (VAS) with “1” indicating the most attractive smile and “5” indicating the least attractive smile.

### 2.3. Statistical Analyses

Data analysis was undertaken using the Statistical Package for Social Science (version 15.0, SPSS Inc., Chicago, Illinois, USA). The mean VAS scores and standard deviation (SD) of each group were calculated. One-way analysis of variance (ANOVA) test was conducted within each group to assess how the groups rated each level of deviation. Significant overall tests were followed by a series of post hoc multiple comparisons (LSD and Bonferroni method) to test hypotheses 1, 2, and 3. LSD was used to detect any significance level between the two closely related professions, orthodontics and general dentist, as this might not be detected with Bonferroni's method which is more conservative. The level of significance was set at *p* < 0.05.

## 3. Results

The mean scores of the photographs were evaluated, and the difference was calculated by using analysis of variance (ANOVA). The mean VAS scores for six different smile esthetic variables as given by orthodontist, dentist, and laypersons as their first choice are shown in Table 1. Analysis by one-way ANOVA revealed *p* value less than 0.05 for all the esthetic variables except crown width. Similarly, comparison of mean VAS scores for gummy smile, midline diastema, crown length, and crown width by one-way ANOVA also demonstrated *p* value less than 0.05 for all the variables except crown width (Table 2). This indicates acceptance of all the smile variables except crown width varies significantly among the three groups. Alterations in crown width do not affect the attractiveness of the smile for orthodontist, general dentist, and laypeople.

### 3.1. Smile Arc

**Figure 7:**
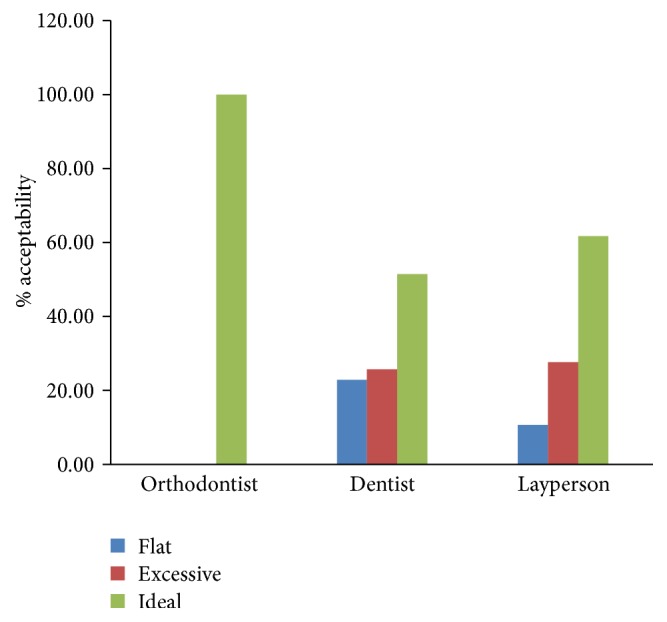
Bar graph representing the smile arc assessment by orthodontists, dentists, and laypersons.

Analyses of VAS scores for smile arc revealed that all the orthodontists (100%) have rated the ideal smile arc (Figure 1(b)) as the most acceptable (Figure 7). A significant proportion of dentists (51.4%) and laypersons (61.7%) had also rated the ideal smile arc as their most preferred one. Next to ideal smile arc, the dentist chose excessive (25.7%) (Figure 1(a)) over flat (22.9%) smile arc (Figure 1(c)). Similarly, laypersons also chose excessive smile arc (27.66%) over flat one (10.64%) (Figure 7). However, dentists were less analytical in accepting deviations in the smile arc than laypersons. On multiple comparison by LSD and Bonferroni's test, a significant difference between orthodontist and dentist (*p* < 0.000, *p* < 0.000) and orthodontist and laypersons (*p* < 0.003, *p* < 0.008) was revealed. However, no significance was established between the dentist and laypersons (Tables 3 and 5). Together, the analysis strongly supported our hypothesis 1 which states that the orthodontists are more critical in analyzing the discrepancies in smile esthetics than other categories in this research.

### 3.2. Buccal Corridors

**Figure 8:**
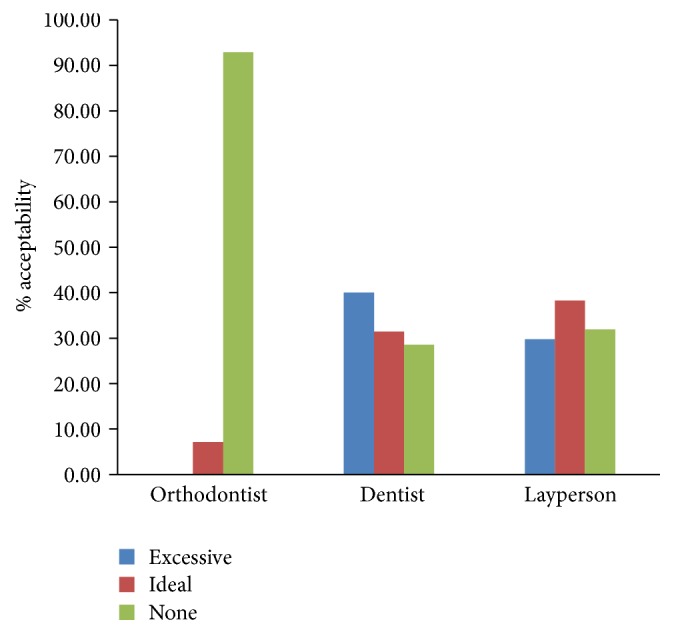
Bar graphs demonstrating the evaluation of the buccal corridor as a smile influencer among orthodontists, dentists, and laypersons.

The orthodontists preferred Hollywood smile (92.86%) the most (Figure 2(c)), followed by ideal buccal corridor (7.14%) (Figure 2(b)). None of them have rated the excessive buccal corridor as their favorite (Figure 8). A large proportion of dentists (40%) had the excessive buccal corridors as their figure of choice followed by ideal buccal corridor (31.43%) and Hollywood smile (28.57%). Approximately, 38% of laypersons chose ideal buccal corridor followed by Hollywood smile (31.91%) and excessive buccal corridor (29.79%) (Figure 8), suggesting that for most dentists and laypersons an excessive buccal corridor was not a deterrent for an attractive smile. Multiple comparison analysis indicated that orthodontist considered the buccal corridor as highly unattractive compared to dentist and laymen (*p* < 0.000) while both dentist and laypeople had comparable views on the presence of buccal corridor, which once again strongly supports our hypothesis 1 (Tables 3 and 5).

### 3.3. Gummy Smiles

**Figure 9:**
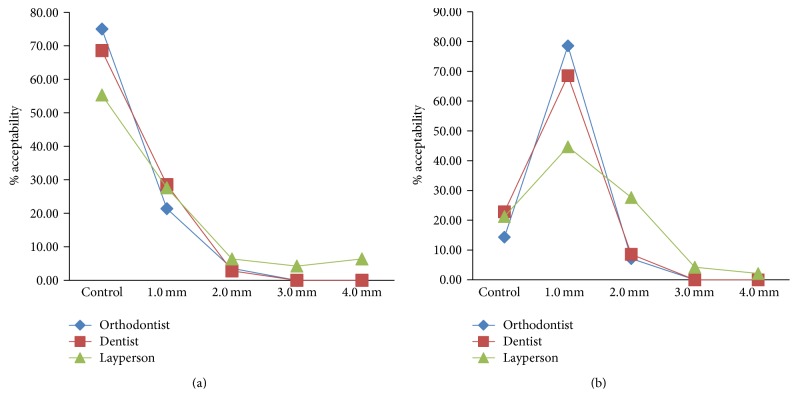
(a) Line graph representing attractiveness of gummy smile as perceived by orthodontists, dentists, and laypersons as their first choice. (b) Line graph representing attractiveness of gummy smile as perceived by orthodontists, dentists, and laypersons as their second choice.

Both orthodontists (75%) and dentists (68.57%) mostly preferred the control image (i.e., 0 gingiva-to-lip distance, Figure 3(a)), followed by 1 mm (21.43% orthodontists and 28.57% dentists) (Figure 3(b)) and 2 mm (4% orthodontists and 2.86% dentists) (Figure 3(c)) of gingiva show as their first choice (Figure 9(a)). In their second choice, the orthodontists (78.57%) and dentists (68.57%) gave preference to 1 mm of gingival show, followed by control image (14.29% orthodontists, 22.86% dentists) and 2 mm of gingival show (7.14% orthodontists, 8.57% dentists) (Figure 9(b)). None of them had rated the images with 3 mm and 4 mm of gingival show as their first or second choice (Figures 9(a) and 9(b)). Similar to orthodontists and dentists, the laypersons also preferred the control image (55.32%), followed by 1 mm (27.66%) of gingiva show. However, they have also rated the gingiva show up to 4.0 mm as attractive (Figures 3(d) and 3(e)). As their second choice, the laypeople preferred 1 mm (44.68%) of gingiva show followed by 2 mm (27.66%), control (21.28%), 3 mm (4.26%), and 4 mm (2.13%) (Figure 9(b)).

Post hoc analysis by LSD method revealed the significant difference that exists between the orthodontist and layperson (*p* = 0.025) as well as between the dentist and layperson (*p* = 0.018) in their first choice (Table 5). In the second choice, the evaluation by dentist and layperson was statistically significant (*p* = 0.017), while for orthodontist and layperson it was very close to significance level (*p* = 0.068) (Table 5). Multiple comparison by Bonferroni's method also indicated that there is no significant difference between the orthodontist and dentist/layperson as well as between the dentist and layperson (*p* > 0.05) (Tables 3 and 4). However, *p* value of 0.053 between the orthodontist and layperson (1st choice, Table 3) and 0.052 between the dentist and layperson (2nd choice, Table 4) indicated the presence of a level very close to significance. Together these results support our second hypothesis which states that laypeople would be less able to discriminate between the levels of discrepancies than the dentists and orthodontists.

### 3.4. Midline Diastema

**Figure 10:**
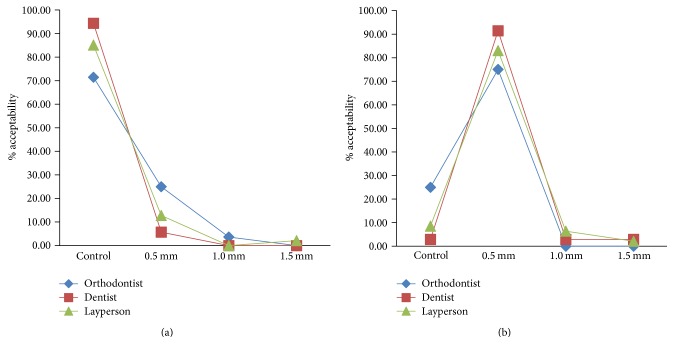
(a) Line graph illustrating acceptance of midline diastema on an attractive smile as first acceptable choice. (b) Line graph representing acceptance of midline diastema on an attractive smile as second choice.

A small amount of space between the maxillary central incisors was not rated as unattractive by any group. All the three groups preferred control image with no midline diastema (71.4% orthodontists, 94.3% dentists, and 85.10% laypersons), followed by the presence of 0.5 mm midline diastema (25% orthodontists, 5.7% dentists, and 12.8% laypersons) (Figures 10(a) and 4). A very small group of orthodontists and laypersons also rated the presence of 1 mm and 1.5 mm of diastema as an attractive smile in their first choice. Similarly, in their second choice, a majority of all the three groups chose 0.5 mm diastema as the first preference for attractive smile (75% orthodontists, 91.42% dentists, and 83% laypersons) (Figure 10(b)).

Multiple comparison by LSD method estimated a significant difference between orthodontist and dentist (*p* = 0.026, *p* = 0.008, resp.) and orthodontist and layperson (*p* = 0.013) (Table 5). Although no statistical significance was observed between all the three groups by Bonferroni's method in the first choice, remarkable difference existed between the second choice made by the orthodontists and dentists (*p* = 0.025) and orthodontists and laypersons (*p* = 0.038) (Table 4). This supports our first hypothesis.

### 3.5. Unilateral Crown Length

**Figure 11:**
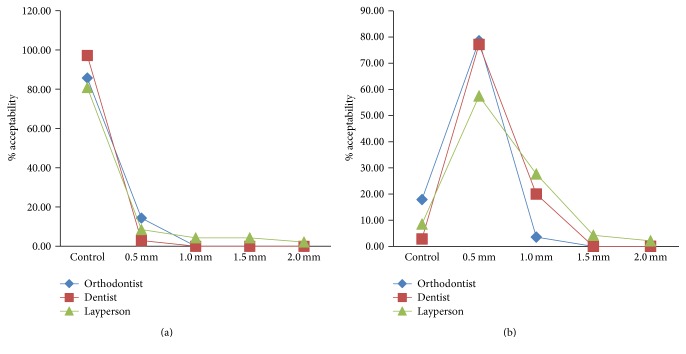
(a) Line graph representing crown length discrepancy by study population as first acceptable choice. (b) Line graph representing crown length discrepancy by study population as second acceptable choice.

All the three groups rated control image as their preferred choice and 0.5 mm as their preferred choice in first and second evaluation, respectively (Figures 11(a) and 11(b)). Both the orthodontists and dentists rated 1 mm of discrepancy as acceptable and nondetectable. However, layperson rated 2 mm of discrepancy as acceptable and nondetectable (Figures 11(a) and 11(b)).

Multiple analysis revealed a significant difference between the orthodontists and laypersons (LSD, *p* = 0.017, *p* = 0.001, Table 5; Bonferroni's method, *p* = 0.003, Table 3), thus, concurring with our hypothesis 2 which states that laypeople are less able to discriminate between the levels of discrepancies than the dentists and orthodontists.

### 3.6. Crown Width

**Figure 12:**
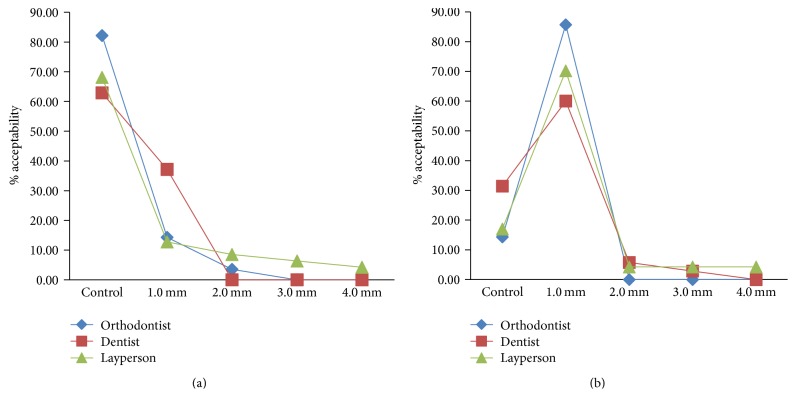
(a) Line graph representing crown width discrepancy by study population as first acceptable choice. (b) Line graph representing crown width discrepancy by study population as second acceptable choice.

The orthodontist group gave the higher ratings for the control group (no discrepancy, 82.14%) first and, then, for the one with a 1 mm (14.29%) discrepancy. A few of them (4%) have also rated 2 mm discrepancy, but their number was of no significance compared to the others (Figure 12(a)). In their second evaluation, the orthodontists gave higher ratings to 1 mm discrepancy (85.71%), followed by control (14.29%), while none have rated 2 to 4 mm discrepancy (Figure 12(b)). Similarly, dentists (62.86%) and laypersons (68.09%) gave first preference to the control image followed by 1 mm of discrepancy (37.14% dentists, 12.77% laypersons) in their first examination. In their second examination, both the groups gave first preference to 0.5 mm discrepancy (60% dentists and 70.21% laypersons), followed by control image (31.43% dentists, 17.02% laypersons). Dentists rated up to 3 mm discrepancy as acceptable and nondetectable in their second evaluation (Figure 12(b)). However, the laypersons could rate up to 4 mm of discrepancy as acceptable and nondetectable in their first and second evaluation (Figures 12(a) and 12(b)).

Multiple comparisons by LSD method could detect a significance level between the dentists and laypersons (*p* = 0.037, Table 5); however, it could not be repeated with Bonferroni's method. Together, the analyses suggest that all the three groups of evaluators were not critical in analyzing the discrepancies in crown width, thus, rejecting our 3rd hypothesis which states that orthodontists are more critical than dentists and so are dentists as compared with laypersons.

## 4. Discussion

Ethnicity strongly influences the acceptance of a smile type in society [6]. A crucial factor for successful outcome of orthodontic treatment is to appreciate the threshold of what society considers acceptable in terms of abnormal smile features. In this study, six common smile variables affecting the beauty of smile, that is, smile arc, buccal corridor, gingival display, midline diastema, crown length, and crown width, were evaluated by the orthodontists, general dentists, and laypeople in Dubai, which is a major city in eastern region of Arabian Peninsula. An interesting aspect of this study is establishing the threshold level of smile variables, which are affected by alteration in length or width, by considering the initial two choices from each group of raters. We presumed that differences in minor alterations (i.e., 0.5 mm or 1 mm) can not be perceived well by three groups of raters; hence, the first two choices would be regarded as pleasant and socially acceptable smile. Considerable group differences for several esthetic discrepancies were observed. Alterations in crown width, however, were not perceived by all the three groups.

Ideal smile arc increases smile attractiveness while a flat smile arc significantly reduces it [11–13]. On the contrary, few studies have reported that smile arc does not contribute to the esthetic value of a beautiful and pleasant smile [14, 15]. Moreover, ethnicity also has a great impact on the preference of smile arc type. While Caucasians chose excessive smile arc, Indians selected ideal smile arc [6]. A remarkable difference existed between Caucasian and Korean populations in choosing smile arc type [16]. Nevertheless, in our study, all the orthodontists and most judges from the other two groups selected the ideal smile arc, suggesting a consensus among Arabic dental professionals and laypeople. However, among the dentists and laypersons, a significant difference was observed in their choice of flat smile arc, wherein laypeople liked flat smile less than the dentists. This is in agreement with the studies of Sarver et al. where orthodontically treated patient could still have an unattractive smile even after the treatment success due to flattening of the smile arc.

Moore et al. reported that a broader smile with minimum buccal corridor was more acceptable and attractive than a narrow smile with large buccal corridors [17]. Orthodontists and laypersons favored smaller corridors than broad corridors [18]. Interestingly, no correlations between smile esthetics and the size of the buccal corridors were found in the study of McNamara et al. [14]. Minimal buccal spaces have been accepted as a feature of attractive smile by various races also [12]. Canadians chose less buccal space than US people [7]. Ker et al. found that narrow buccal corridors were more favorable for laypersons in the west coast compared to the Midwest and east coast in the USA, indicating the presence of regional differences [19]. Koreans, Japanese, Caucasians, and Afrodescendants also preferred narrow or medium buccal corridors [20, 21]. In Indian population, orthodontist chose less buccal space as an element for a pleasant smile while for laypeople buccal corridor of any width was not required for pleasant smile [15]. Agreeing with Divyaroop Rai et al., in the current study, the orthodontists from eastern Arabic region concluded that smiles were more attractive when the buccal corridor was absent (Hollywood smile) or when there was a minimal medium-broad ideal buccal corridor. However, the general dentists and laypersons were not critical of an excessive buccal corridor, thus, confirming that the buccal corridor alone is not a critical influence of smile attractiveness.

Extent of gingival display affecting smile esthetics is variable [22]. The attributes of a youthful smile include a full display of the maxillary incisor crowns, with 1-2 mm of gingiva show [23]. Excessive display of the gingiva known as “gummy smile” can render a smile unattractive. A gingival display of up to 1 mm was accepted to be attractive by Saudi dentists and laypeople [9]. In both Caucasians and Afro-Brazilian, gingival display up to 1 mm was considered esthetic while ≥3 mm was considered unaesthetic [24]. However, for Americans, the threshold of a gingival display was 4 mm [25]. The present study indicated that a display of up to 2 mm was scored as attractive by the orthodontists, whereas laypersons opined that a display of up to 4 mm was also acceptable. Our result demonstrated that eastern Arabic region people have more tolerance for gingival display compared to central Arabic region as well as Caucasians and Afro-Brazilians. This can be attributed to our methodological approach wherein the threshold was based on two ratings for each photograph.

The presence of a large midline diastema negatively affects smile esthetics, and such persons are considered to be socially less successful [22, 26]. In Indian population, diastema was considered unaesthetic at a threshold level of 1.5 mm, whereas in Africans midline diastema was considered esthetic provided the width was within 2 to 3 mm [27, 28]. Saudi dentists and laypeople considered small midline diastema as unattractive [9]. In this study, the threshold for unattractiveness for midline diastema was found to be less in orthodontists and dentists compared to the layperson, which is in accord with the American populations [22]. Orthodontists and dentists rated the diastema unattractive when it was more than 1 mm wide, whereas for the laypersons the threshold was found to be 1.5 mm suggesting a diastema less than 1 mm that is not objectionable for people of eastern Arabic region.

Recent studies established that American laypeople did not distinguish asymmetric crown length unless one crown was 1.5–2.0 mm shorter than the other [22, 29]. Unilateral crown length shortening of greater than 1.5 mm compared to contralateral tooth was perceived as equally unaesthetic by all three groups of Indian respondents [28]. Compared to laypeople, Saudi dentists gave lower ratings to a crown length discrepancy of >2 mm. This study results also corroborated that 2 mm is the limit of acceptability for this variable by both central and eastern Arabic laypersons. Asymmetric alterations in teeth appeared unattractive to both the dental professionals and laypersons [22]. Asymmetrical alterations in the crown width of the lateral incisor showed a threshold level of 1.5 mm in Indians [28]. Compared to laypeople, the Saudi dentists gave lower ratings to crown width discrepancy of >2 mm. However, this was true for only orthodontists in our study. A mesiodistal dimension of 2.0 mm narrower than the ideal lateral incisor crown width was required before it was rated significantly less attractive by orthodontists while for dentists the threshold was 3.0 mm. A 4.0-mm proportional narrowing of mesiodistal width was necessary for laypersons to rate it noticeably less attractive. Findings from this study suggested that the clinician should initially measure the difference in width between the maxillary lateral incisors before planning any treatment. If the discrepancy is 1 mm or less, restoration is probably not necessary, because it will likely not be recognized. However, if the difference is 2 mm or greater, the narrower tooth should be restored.

All the analysis was performed on the female smile which is one of the limitations of the present study. Geron and Atalia [30] have shown that the gender of the smile image affects smile attractiveness, thus, biasing our results. Moreover, another limitation of this study is that the socioeconomic status of the laypersons was not considered, which may have affected the results.

## 5. Conclusion

The degree of perception of smile esthetics to be attractive varies between Arabic orthodontists, dentists, and laypersons. Arabic orthodontists were more analytical in judging variables like smile arc and buccal corridor. However, these variables did not affect the general dentists and laypersons much. The majority of Arabic dental professionals and laypeople preferred either complete absence or 0.5 mm of alterations in the gingival display, midline diastema, and crown length while smiling. Crown width did not form a critical element of the pleasant and beautiful smile in Arabic population. Hence, Arabic orthodontists should keep a note of the amount of modifications to be made in the smile arc and buccal corridor space that is acceptable to the layperson while an orthodontic treatment and the patient's perception of smile esthetics should be given importance before any treatment is intended.

## Figures and Tables

**Table 1:** Comparison of mean esthetic scores of different smile variables as evaluated by the study populations.

Smile variables	Orthodontist mean ± SD (*n* = 28)	Dentist mean ± SD (*n* = 35)	Laypersons mean ± SD (*n* = 47)	*F* value^#^	*p* value
Smile arc	2 ± 0.000	1.29 ± 0.825	1.53 ± 0.680	9.713	0.000^*∗∗∗*^
Buccal corridor	1.93 ± 0.262	0.89 ± 0.832	0.801 ± 0.114	18.617	0.000^*∗∗∗*^
Gummy smile (1st acceptable choice)	1.29 ± 0.535	1.34 ± 0.539	1.78 ± 1.141	3.968	0.022^*∗*^
Midline diastema (1st acceptable choice)	1.32 ± 0.548	1.06 ± 0.236	1.18 ± 0.527	2.542	0.083
Crown length (1st acceptable choice)	1.14 ± 0.356	1.03 ± 0.169	1.37 ± 0.906	3.098	0.049^*∗*^
Crown width (1st acceptable choice)	1.21 ± 0.499	1.37 ± 0.490	1.63 ± 1.131	2.433	0.093

SD: standard deviations, ^#^one-way ANOVA, ^*∗*^ *p* ≤ 0.05, and ^*∗∗∗*^ *p* ≤ 0.001.

**Table 2:** Comparison of mean esthetic scores of different smile variables as evaluated by the study populations in their second preference.

Smile variables	Orthodontist mean ± SD (*n* = 28)	Dentist mean ± SD (*n* = 35)	Laypersons mean ± SD (*n* = 47)	*F* value^#^	*p* value
Gummy smile	1.93 ± 0.466	1.86 ± 0.550	2.24 ± 0.925	3.404	0.037^*∗*^
Midline diastema	1.75 ± 0.441	2.06 ± 0.416	2.02 ± 0.478	4.293	0.016^*∗*^
Crown length	1.86 ± 0.448	2.17 ± 0.453	2.37 ± 0.809	5.776	0.004^*∗∗*^
Crown width	1.86 ± 0.356	1.86 ± 0.550	2.24 ± 0.925	1.456	0.238

SD: standard deviations, ^#^one-way ANOVA, ^*∗*^ *p* ≤ 0.05, and ^*∗∗*^ *p* ≤ 0.01.

**Table 3:** Intragroup comparison of esthetic scores by Bonferroni's method (1st acceptable choice).

Smile variables	Group comparison	*p* value
Smile arc	Orthodontist × dentist	0.000^*∗∗∗*^
	Dentist × layperson	0.267
	Orthodontist × layperson	0.008^*∗∗*^

Buccal corridor	Orthodontist × dentist	0.000^*∗∗∗*^
	Dentist × layperson	0.816
	Orthodontist × layperson	0.000^*∗∗∗*^

Gummy smile (1st choice)	Orthodontist × dentist	1.000
	Dentist × layperson	0.074
	Orthodontist × layperson	0.053

Midline diastema (1st choice)	Orthodontist × dentist	0.079
	Dentist × layperson	0.658
	Orthodontist × layperson	0.635

Crown length (1st choice)	Orthodontist × dentist	1.000
	Dentist × layperson	0.052
	Orthodontist × layperson	0.413

Crown width (1st choice)	Orthodontist × dentist	1.000
	Dentist × layperson	0.483
	Orthodontist × layperson	0.111

^*∗∗*^ *p* ≤ 0.01, and ^*∗∗∗*^ *p* ≤ 0.001.

**Table 4:** Intragroup comparison of esthetic scores by Bonferroni's method (2nd acceptable choice).

Smile variables	Group comparison	*p* value
Gummy smile (2nd choice)	Orthodontist × dentist	1.000
	Dentist × layperson	0.052
	Orthodontist × layperson	0.204

Midline diastema (2nd choice)	Orthodontist × dentist	0.025^*∗*^
	Dentist × layperson	1.000
	Orthodontist × layperson	0.038^*∗*^

Crown length (2nd choice)	Orthodontist × dentist	0.159
	Dentist × layperson	0.496
	Orthodontist × layperson	0.003^*∗∗*^

Crown width (2nd choice)	Orthodontist × dentist	1.000
	Dentist × layperson	0.379
	Orthodontist × layperson	0.614

^*∗*^ *p* ≤ 0.05. ^*∗∗*^ *p* ≤ 0.01.

**Table 5:** Intragroup comparison of esthetic scores by LSD method.

Smile variables	Group comparison	*p* value
Smile arc	Orthodontist × dentist	0.000^*∗∗∗*^
	Dentist × layperson	0.089
	Orthodontist × layperson	0.003^*∗∗*^

Buccal corridor	Orthodontist × dentist	0.000^*∗∗∗*^
	Dentist × layperson	0.272
	Orthodontist × layperson	0.000^*∗∗∗*^

Gummy smile (1st choice)	Orthodontist × dentist	0.793
	Dentist × layperson	0.025^*∗*^
	Orthodontist × layperson	0.018^*∗*^

Gummy smile (2nd choice)	Orthodontist × dentist	0.698
	Dentist × layperson	0.017^*^
	Orthodontist × layperson	0.068

Midline diastema (1st choice)	Orthodontist × dentist	0.026^*∗*^
	Dentist × layperson	0.219
	Orthodontist × layperson	0.212

Midline diastema (2nd choice)	Orthodontist × dentist	0.008^*∗∗*^
	Dentist × layperson	0.713
	Orthodontist × layperson	0.013^*∗*^

Crown length (1st choice)	Orthodontist × dentist	0.478
	Dentist × layperson	0.138
	Orthodontist × layperson	0.017^*∗*^

Crown length (2nd choice)	Orthodontist × dentist	0.053
	Dentist × layperson	0.165
	Orthodontist × layperson	0.001^*∗∗*^

Crown width (1st choice)	Orthodontist × dentist	0.460
	Dentist × layperson	0.037^*∗*^
	Orthodontist × layperson	0.111

Crown width (2nd choice)	Orthodontist × dentist	0.880
	Dentist × layperson	0.126
	Orthodontist × layperson	0.205

^*∗*^ *p* ≤ 0.05, ^*∗∗*^ *p* ≤ 0.01, and ^*∗∗∗*^ *p* ≤ 0.001.
